# *Vagococcus lutrae* Isolation in a Cat with Feline Urological Syndrome in Italy: A Case Report

**DOI:** 10.3390/microorganisms13092020

**Published:** 2025-08-29

**Authors:** Daniela Averaimo, Sabrina Vanessa Patrizia Defourny, Alessandra Alessiani, Marco Rulli, Alexandra Chiaverini, Marco Di Domenico, Iolanda Mangone, Cinzia Pompilii, Vanessa Piersanti, Roberta Giancristofaro, Lucilla Ricci, Antonio Petrini

**Affiliations:** 1 Istituto Zooprofilattico Sperimentale dell’Abruzzo e Molise “G. Caporale”, 64100 Teramo, Italy; d.averaimo@izs.it (D.A.); a.alessiani@izs.it (A.A.); m.rulli@izs.it (M.R.); a.chiaverini@izs.it (A.C.); m.didomenico@izs.it (M.D.D.); i.mangone@izs.it (I.M.); c.pompilii@izs.it (C.P.); l.ricci@izs.it (L.R.); a.petrini@izs.it (A.P.); 2 Dipartimento di Medicina Clinica, Sanità Pubblica, Scienze della Vita e Dell’ambiente, Università degli Studi dell’Aquila, 67100 L’Aquila, Italy; vanessa.piersanti212@gmail.com; 3 Clinica Veterinaria San Francesco, 66034 Lanciano, Italy; robertagiancristofaro@gmail.com

**Keywords:** *Vagococcus lutrae*, cat, AMR, case report, emerging pathogens

## Abstract

*Vagococcus lutrae* is an emerging pathogen that can cause severe disease, especially in immunocompromised patients. Unlike *Vagococcus fluvialis*, which is recognized as a human and animal pathogen, there are few reports of *V. lutrae* from human and animal infections. In humans, it has been reported in patients with severe skin lesions and bloodstream infections. In veterinary medicine, *V. lutrae* was accidentally isolated from a Eurasian otter and a largemouth bass, and only once from the genitourinary tract of a pig with a urinary tract infection. However, the prevalence may be underestimated due to difficulties in identification using traditional methods. In addition, *V. lutrae* could be a carrier of resistance genes and contribute to the spread of AMR. A neutered male cat with feline urological syndrome underwent urethrostomy surgery due to serious problems with dysuria and urolithiasis that could not be resolved through catheterizations. Urine culture revealed the presence of *Vagococcus lutrae*. The strain showed resistance genes against aminoglycoside, lincosamide, streptogramin a and b, pleuromutilin, macrolide, tetracycline, oxazolidinone, and amphenicol classes. We report the first isolation of *V. lutrae* from the urinary tract of a cat.

## 1. Introduction

*Vagococcus* spp. has been described as a potential emerging bacterial risk [1] with a zoonotic feature. This pathogen is a Gram positive, catalase-negative, motile, or non-motile bacterium belonging to the *Enterococcaceae* family, and is phylogenetically closely related to the genus *Enterococcus*, based on 16S rRNA gene sequences [2]. Unlike *V. fluvialis*, which is well documented as a pathogen in both human [3] and veterinary medicine [4], *V. lutrae*—one of the nine recognized species within the genus—is rarely reported as a causative agent of infection. It was first isolated from multiple organs (blood, liver, lungs, and spleen) of a Eurasian otter (*Lutra lutra*) found dead following a vehicular collision in the UK [5]. In that case, it was not possible to associate *V. lutrae* with the cause of death. Another report describes its isolation from the intestine of a largemouth bass (*Micropterus salmoides*) in the USA [6]. A third case involved its recovery from the genitourinary tract of a diseased pig presenting with a urinary tract infection [7].

To the best of our knowledge, only one previous study has reported the presence of a *Vagococcus* species in felines. In that case, a strain of *V. fluvialis* was isolated from tonsillar tissue as well as from a pooled sample of spleen and liver collected during the necropsy of a cat exhibiting hepatocellular icterus [4].

To date, only four human cases have been reported: two involving severe skin lesions [8,9] and two a bloodstream infection, one of which accompanied a history of chronic kidney disease and prostate cancer [10,11].

The rarity of reports of *V. lutrae* as a cause of infection may be due to the challenges in identification using traditional methods and/or the likeness to other cocci belonging to neighbor genera [12].

Importantly, animals—especially cats and dogs—might serve as a reservoir of bacteria with resistance genes, due to the increasing use of raw meat-based diets (RMBDs) as an alternative to commercially available pet foods [1,2,12,13]. One of the major problems with RMBDs is that the ingredients, including meat, are neither cooked nor pasteurized, thus representing a potential infection risk for both animals and their owners [13]. *V. lutrae* could act as a donor of antibiotic resistance determinants, contributing to the spread of antimicrobial resistance (AMR), which is one of the main public health challenges today. Here, we report the first case of the isolation of *V. lutrae* from the urinary tract of a domestic shorthair cat in Italy.

## 2. Materials and Methods

### 2.1. Case Presentation

A one-year-old neutered male shorthair cat with a history of dysuria and urolithiasis, secondary to severe feline urological syndrome, was presented in March 2024 to the San Francesco Veterinary Clinic in Lanciano, Chieti, Italy. The cat lived in a colony, had a commercial diet, and was FIV and FeLV negative, but not vaccinated against the major feline diseases.

On clinical examination, the primary finding was dysuria with the passage of mucous urine with abundant necrotic fragments, likely due to sloughing of the bladder mucosa. Ultrasound showed a thickened, edematous bladder wall with areas of gas infiltration. The bladder contents were non-homogeneous hyperechoic, with floating hyperechoic formations of various sizes, sometimes with a shadow cone. Urinalysis revealed high pH values (>6.5), hematuria, proteinuria, and pyuria, with SG > 1030. Sediment analysis reported numerous struvite crystals as well as leukocytes, erythrocytes, and amorphous material suggestive of mucus and fragments of necrotic bladder mucosa.

After two catheterizations failed to resolve the issue due to obstruction, the cat was anesthetized and an urethrostomy was performed. Urine was collected via a sterile catheter after disinfecting the area surrounding the stoma with povidone-iodine solution and alcohol. The sample was submitted for urine culture to the Istituto Zooprofilattico Sperimentale dell’Abruzzo e del Molise (IZSAM), Teramo, Italy. After surgery, amoxicillin/clavulanate therapy was initiated; however, given the worsening clinical condition, the cat was subjected to euthanasia.

### 2.2. Isolation, Identification and Characterization of V. lutrae

**Figure 1:**
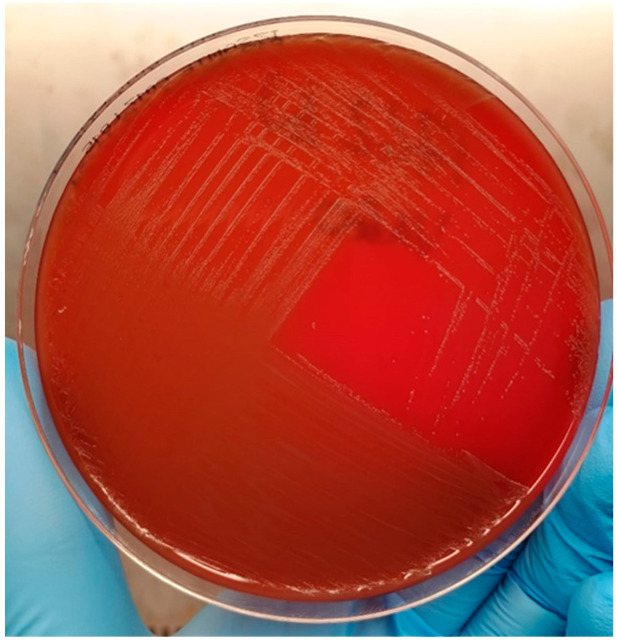
*Vagococcus lutrae* on sheep blood agar after 48 h of incubation.

IZSAM performed a culture test on the urine sample using direct plating on sheep blood agar, chocolate agar, Gassner agar, mannitol salt agar, and Sabouraud dextrose agar (Microbiol srl, Cagliari, Italy) at 37 °C ± 1 °C under aerobic conditions (Figure 1). The Gram stain (Merck KGaA, Darmstadt, Germany) was carried out on single colonies after colony growth and subculturing, according to the manufacturer’s instructions. The identification was performed by the Maldi Biotyper^®^ system using the MALDI Biotyper Compass software version 4.1 (MALDI-TOF Biotyper^®^, Bruker Daltonics Gmbh & Co. KG; Bremen, Germany). The identification score was considered acceptable when ≥1.7 according to the manufacturer’s instructions.

Antimicrobial resistance was evaluated using broth microdilution with COMPGP1F^®^ plates and the Sensititre SWIN^®^ Software System version 3.4 (ThermoFisher Scientific, Milano, Italy) according to the manufacturer’s instructions. The antimicrobials tested were: amikacin (16–32 µg/mL), amoxicillin/clavulanic acid (0.25/0.12–8/4 µg/mL), ampicillin (0.25–8 µg/mL), cefazolin (2–4 µg/mL), cefovecin (0.06–8 µg/mL), cefpdoxime (2–8 µg/mL), cephalothin (2–4 µg/mL), chloramphenicol (8–32 µg/mL), clindamycin (0.5–4 µg/mL), doxyciclyne (0.12–0.5 µg/mL), enrofloxacin (0.25–4 µg/mL), erythromycin (0.25–4 µg/mL), gentamicin (4–16 µg/mL), imipenem (1–4 µg/mL), marbofloxacin (1–4 µg/mL), minocycline (0.5–2 µg/mL), nitrofurantoin (16–64 µg/mL), oxacillin +2% NaCl (0.25–2 µg/mL), penicillin (0.06–8 µg/mL), pradofloxacin (0.25–2 µg/mL), rifampin (1–2 µg/mL), tetracycline (0.25–1 µg/mL), trimethoprim/sulfamethoxazole (2/38–4/76 µg/mL), and vancomycin (1–16 µg/mL).

DNA extraction was performed using the Maxwell^®^ RSC Genomic DNA Kit on Maxwell^®^ RSC Instrument (Promega, Milano, Italy), and DNA quantity and quality were evaluated with a Quantus™ Fluorometer (Promega, Milano, Italy) according to the manufacturer’s instructions. Whole genome sequencing (WGS) was performed as reported in Bosica et al., 2023 [14]. The largest contigs were blasted on GenBank (NCBI). Trimmed reads were then mapped using Bowtie2 [15] against the genome founded on Bowtie2 Software Version 2.5.4. AMR gene analysis was performed using ResFinder (accessed on August 2024) [16]. The virulence potential of this pathogen was checked using VirulenceFinder 2.0 (accessed on August 2024) [17].

## 3. Results

After 48 h of incubation on blood and chocolate agar, pure, smooth, small, grayish-white colonies with α-hemolysis were isolated (Figure 1). Gram stain showed Gram-positive cocci. The colonies were identified by MALDI-TOF as *Vagococcus lutrae* with a high-confidence identification value of 2.11.

Antimicrobial test results were assessed using CLSI VET01S ED7:2024 as a first choice, or CLSI M100 ED35:2025 and EUCAST Clinical Breakpoints (v15.0, accessed on 4 August 2025) (www.eucast.org) [18] when the tested molecules were not listed in the previous one [19,20]. References for *Enterococcus* spp. were used for the interpretation of the results because *Vagococcus* spp. was not listed. Table 1 shows the antimicrobials for which an activity range could be defined in the previously listed databases. The table shows only usable results. In fact, for most of the molecules, no interpretation values are available in any reference database, consequently they would be considered as ‘not interpretable’.

Regarding molecular characterization, the de novo assembly resulted in 2,026,063 bp combined in 94 contigs. The largest contigs were blasted on GenBank and the best record was the *Vagococcus lutrae* strain BN31 chromosome, complete genome (CP081833.1). The reference-guided assembly resulted in a unique contig of 2,028,588 bp with a mean coverage depth of 157×. Raw data obtained in this study were submitted in NCBI under the Biosample accession SAMN46358844. Several resistance genes were detected against the aminoglycoside, lincosamide, streptogramin a and b, pleuromutilin, macrolide, tetracycline, oxazolidinone, and amphenicol classes (Table 2). No virulence gene was found.

## 4. Discussion and Conclusions

Since its first description in 1947 [21] and subsequent classification as a new genus by Collins in 1989 [2], *Vagococcus* spp. have been identified in various sources such as animal (including insects), human, and environmental matrices [22]. Different species of *Vagococcus* (*V. salmoninarum* and *V. fluvialis*) have been found on the rind of raw and pasteurized milk cheeses [23].

In all of the human cases, the source of infection remained unclear. In the first one [8], the authors hypothesized a foodborne origin, linked to the ingestion of fish and/or other seafood. In the second case [9], bacteremia was thought to be secondary to a skin infection, but they were unable to trace the origin as no consumption of cheese or seafood and no contact with pets or contaminated water was reported. In the last two reported, the authors made no hypotheses about the origin of the infection [10,11]. Due to the limited information in the literature, it was also not possible in our case to correlate the clinical signs or the origin of infection with this bacterium. In this case, as the cat received commercial food, there was no risk associated with the consumption of RMBDs. However, since the cat belonged to a feline colony, it may have contracted the infection by consuming contaminated food waste, given that the presence of *Vagococcus* spp. has been reported in marine animals and milk products [10,11,12,13,14,15,16,17,18,19,20,21,22,23]. The infection might also have been transmitted through contact with soil, as *V. lutrae* has been reported in the intestine of pigs and otter. In this context, we hypothesize that rodents may also play a role in the transmission of the bacterium given that rodents are known to be reservoir hosts for many zoonotic diseases [24]. However, we can only speculate on these routes of infection, as unfortunately no environmental sampling or epidemiological studies have been conducted to support these hypotheses. The use of environmental sampling in future cases could likely help to better understand the origin of the infection and confirm or disprove this hypothesis.

Few cases of *V. lutrae* have been reported to date; however, the prevalence could be underestimated because identification with traditional methods is tricky, in particular when distinguishing it from other bacteria belonging to related genera like *Enterococcus* [8,9,10,11,12]. Sheep blood agar and chocolate agar exhibited significantly higher growth rates under aerobic conditions, displaying purity in the results. The new technologies, MALDI-TOF coupled with WGS, enabled a high-confidence identification. Furthermore, WGS enabled the genomic characterization of this pathogen in terms of virulence and AMR prediction, representing a powerful tool for analyzing emerging pathogens of public health concern [14,15,16,17,18,19,20,21,22,23,24,25,26].

As suggested by previous work [11], *Vagococcus* spp. can be considered a pathogen only when isolated in pure culture from a sample, while it can be categorized as an opportunistic bacterium if isolated in a polymicrobial flora. In our case, the culture of the urine sample tested positive only for *V. lutrae*.

After surgery, and before the susceptibility test results, an antibiotic therapy was administered to the cat. Values obtained from the antimicrobial susceptibility testing (AST) by broth microdilution, indicated that the strain was susceptible to the penicillin group, as reported by Altintas et coll. [10].

The aminoglycosides class was not interpretable. However, it is known that enterococci are intrinsically resistant to the aminoglycosides, when used in monotherapy. Analysis of the AMR gene confirmed the presence of the aminoglycoside resistance gene *aph(3*′*)-III*. AST showed sensitivity to vancomycin, which often causes resistance problems in *Enterococcus* spp.

Based on known breakpoints [19,20], tetracycline’s MIC values were classified as not interpretable, but AMR gene analysis revealed the presence of the tetracycline resistance gene tet(L). *V. lutrae* strain showed also the linezolid resistance gene *optrA*, as reported in previously study [13].

AST indicated that the strain was susceptible to cefazolin; however, CLSI guidelines highlight that for *Enterococcus* spp., cephalosporins, clindamycin, and trimethoprim/sulfamethoxazole may demonstrate activity in vitro but are not clinically effective, and thus should not be reported as susceptible. More generally, it can be said that the high number of resistance genes, known as elements characterised by their ability to transfer between bacterial populations, has allowed this strain of *V. lutrae* to accumulate a large number of resistance genes, making it extremely dangerous in terms of antimicrobial resistance.

Our work represents the first report of isolation of *V. lutrae* from the urinary tract in a cat, while also confirming the presence of resistance genes in the strain. Even if *V. lutrae* is rarely reported, it could play a role in transmission of resistance genes to other pathogens. Notably, in the gastrointestinal or skin habitat *Vagococcus*, which is phylogenetically close to *Enterococcus* spp., can easily acquire resistance genes from other commensal organisms, or can transfer them to others pathogenic bacteria. Therefore, to prevent the development of antimicrobial resistance, we emphasize the importance of performing AST before starting any antibiotic therapy. Furthermore, it would be advisable to consider *V. lutrae* as a potential pathogen in patients with urinary tract infections or skin lesions—particularly in cases of antibiotic treatment failure—and to carry out a thorough culture-based diagnostic evaluation.

## Figures and Tables

**Table 1:** Results of antimicrobic susceptibility test by broth microdilution of the *Vagococcus lutrae* strain.

Class	Antimicrobial	Results (µg/mL)	Interpretation
Carbapenems	Imipenem ^3^	≤1	S
Glycopeptides	Vancomycin ^1^	≤1	S
Penicillinase-labile penicillins	Ampicillin ^1^	≤0.25	S
	Penicillin ^2^	≤0.06	S
β-lactam combination agents	Amoxicillin-clavulanate ^1^	≤0.25	S
Phenicols	Chloramphenicol ^1^	>32	R

Interpretation based on CLSI VET01S ED7:2024 (designated 1), CLSI M100 ED35:2025 (designated 2), and EUCAST (designated 3). R = resistant, S = sensible.

**Table 2:** Results of AMR gene analysis.

Class	Gene
Aminoglycoside	*aph(3′)-III*
Lincosamide	*lsa(E), lnu(B), erm(B), erm(A)*
Streptogramin a	*lsa(E)*
Pleuromutilin	*lsa(E)*
Macrolide	*erm(B), erm(A)*
Tetracycline	*tet(L)*
Streptogramin b	*erm(B), erm(A)*
Oxazolidinone	*optrA*

## Data Availability

All data are available at the NIH-PMC website.

## Abbreviations

The following abbreviations are used in this manuscript:

AMR	antimicrobial resistance
RMBDs	raw meat-based diets
FIV	feline immunodeficiency virus
FeLV	feline leukemia virus
pH	potential of hydrogen
SG	specific gravity
IZSAM	Istituto Zooprofilattico Sperimentale dell’Abruzzo e del Molise
MALDI-TOF	matrix-assisted laser desorption/ionization time-of-flight
MIC	minimal inhibitory concentration
WGS	whole genome sequencing
